# Minimally Invasive Management of Bile Leak Following Displaced Percutaneous Transhepatic Biliary Stent

**DOI:** 10.7759/cureus.22456

**Published:** 2022-02-21

**Authors:** Sanjeyan J, Sreekanthan Gobishangar

**Affiliations:** 1 University Surgical Unit, Teaching Hospital Jaffna, Jaffna, LKA; 2 Department of Surgery, Faculty of Medicine, University of Jaffna, Jaffna, LKA

**Keywords:** interventional radiology, laparoscopic management, biliary leak, bile duct stricture, percutaneous transhepatic biliary stenting

## Abstract

Biliary endoprosthesis plays a crucial role in the management of patients with obstructive jaundice. However, a biliary leak is a life-threatening complication of this procedure. A 52-year-old otherwise healthy man presented with obstructive jaundice and was found to have a stricture at the confluence of the right and left hepatic ducts, which was managed with the placement of an uncovered self-expanding metallic stent. He rapidly deteriorated, and an active bile leak in the peritoneum due to stent displacement through the liver was discovered, which was successfully managed in a minimally invasive manner via laparoscopy. The extrahepatic part of the metallic stent was cut and removed, the peritoneum was washed out, and multiple drains were placed. The patient improved clinically, and his biochemical parameters returned to normal.

## Introduction

Patients with obstructive jaundice due to biliary drainage obstruction are better managed with biliary endoprosthesis, which can be placed during endoscopic retrograde cholangiopancreatography (ERCP) or percutaneous transhepatic biliary stenting [1,2]. Although a biliary leak is a life-threatening complication of the procedure [3], the incidence of bile leak is higher with percutaneous transhepatic biliary stenting than ERCP stenting, especially when the stent is displaced resulting in a continuous leak. Biliary leaks are traditionally managed by open surgery [4]. Here, we report a case of a persistent bile leak into the peritoneum and subsequent sepsis due to a displaced metallic biliary stent that was successfully managed in a minimally invasive manner by laparoscopic excision of the extrahepatic part of the metallic stent, peritoneal washout, and multiple drain placement. This is a rare complication, and no previous cases have been reported.

## Case presentation

A 52-year-old apparently well man presented with intermittent pain in the right hypochondrium, loss of appetite, and significant weight loss over a one-month period. Further, he reported yellowish discoloration of the sclera, dark urine, pale stools, and pruritus during the past week. On clinical examination, he was ill-looking, icteric, and had mild pallor; however, his abdominal examination was unremarkable. His heart rate was 58 beats per minute, and his blood pressure was 130/70 mmHg. Other system examinations were unremarkable.

His total bilirubin, direct bilirubin, and alkaline phosphatase levels were 462.9 µmol/L, 438.4 µmol/L, and 502 U/L, respectively, which supported the diagnosis of obstructive jaundice. His other hematological and biochemical parameters were as follows: white blood cell count, 8.51 × 10^9^/L; hemoglobin, 9.6 g/dL; platelet count, 252 × 10^9^/L; serum creatinine, 78 mmol/L; alanine aminotransferase, 283 U/L; aspartate aminotransferase, 106 U/L; albumin, 28 g/L; globulin, 28 g/L; and international normalized ratio, 1.2.

Transabdominal ultrasonography revealed uncomplicated gallstones and intrahepatic duct dilatation with a normal common bile duct (CBD). Contrast-enhanced computerized tomography (CECT) images showed abnormally dilated and tortuous intrahepatic ducts in the liver parenchyma that converged into the hilum. However, the common hepatic duct was not visualized at its confluence. The gallbladder was moderately dilated and contained multiple calculi and one hyperdense calculus in its neck measuring 8.4 × 8.9 mm. Hyperdense sludge was also observed in the gallbladder. The pancreatic duct was not dilated. The CECT images of the pancreas were normal. No significant lymph nodes were observed in the porta hepatis or para-aortic regions. The liver was not enlarged, its surface appeared smooth, and there was no evidence of capsular retraction. No tumor or neoplasm was noted at the confluence of the hepatic duct. Enhancement was not delayed in the expected region, even at 120 s.

**Figure 1:**
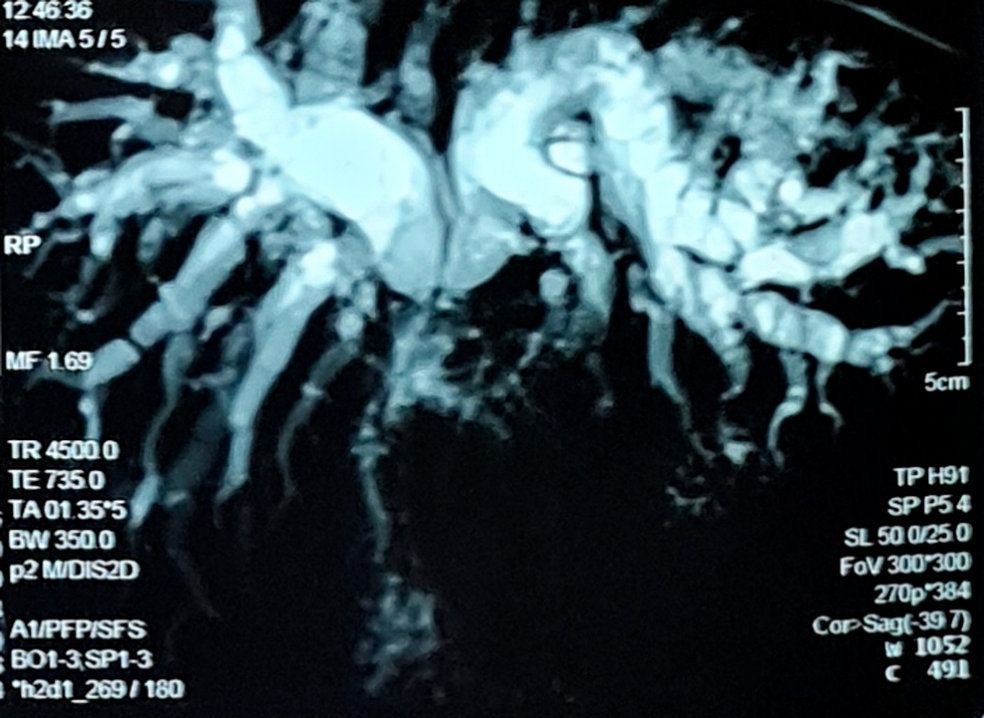
MRCP image showing dilated biliary system up to the confluence of hepatic ducts. MRCP: magnetic resonance cholangiopancreatography

The magnetic resonance cholangiopancreatography images showed a narrowing of the proximal hepatic duct with intrahepatic bile duct dilatation, normal caliber CBD, gallbladder stones, and no masses in the pancreas or duodenum (Figure 1).

**Figure 2:**
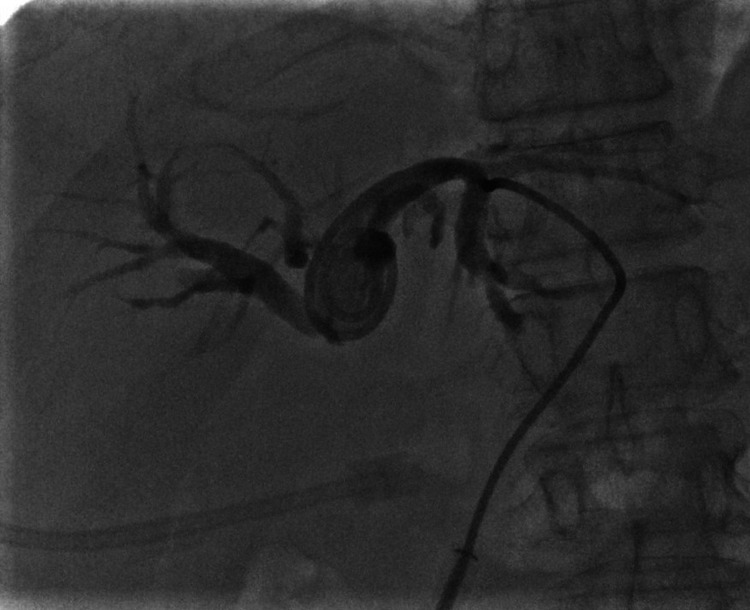
Fluoroscopy-guided EBD from the right hepatic duct. EBD: external biliary drainage

ERCP and stenting were attempted but could not be completed due to a tight stricture, which did not allow the guidewire to pass through. Unfortunately, the patient developed coronavirus disease 2019 (COVID-19) pneumonia in the subsequent days and was transferred to the COVID-19 specialty ward. As a temporary life-saving measure, fluoroscopy-guided external biliary drainage (EBD) from the right hepatic duct was established (Figure 2) along with administration of intravenous (IV) antibiotics (750 mg cefuroxime, every eight hours, for five days).

His symptoms started to improve, and his bilirubin levels decreased to 60 mmol/L; however, the patient had a persistently high output through the EBD (750 to 1,300 mL per day) for 20 days. Fortunately, he recovered from COVID-19 pneumonia.

**Figure 3:**
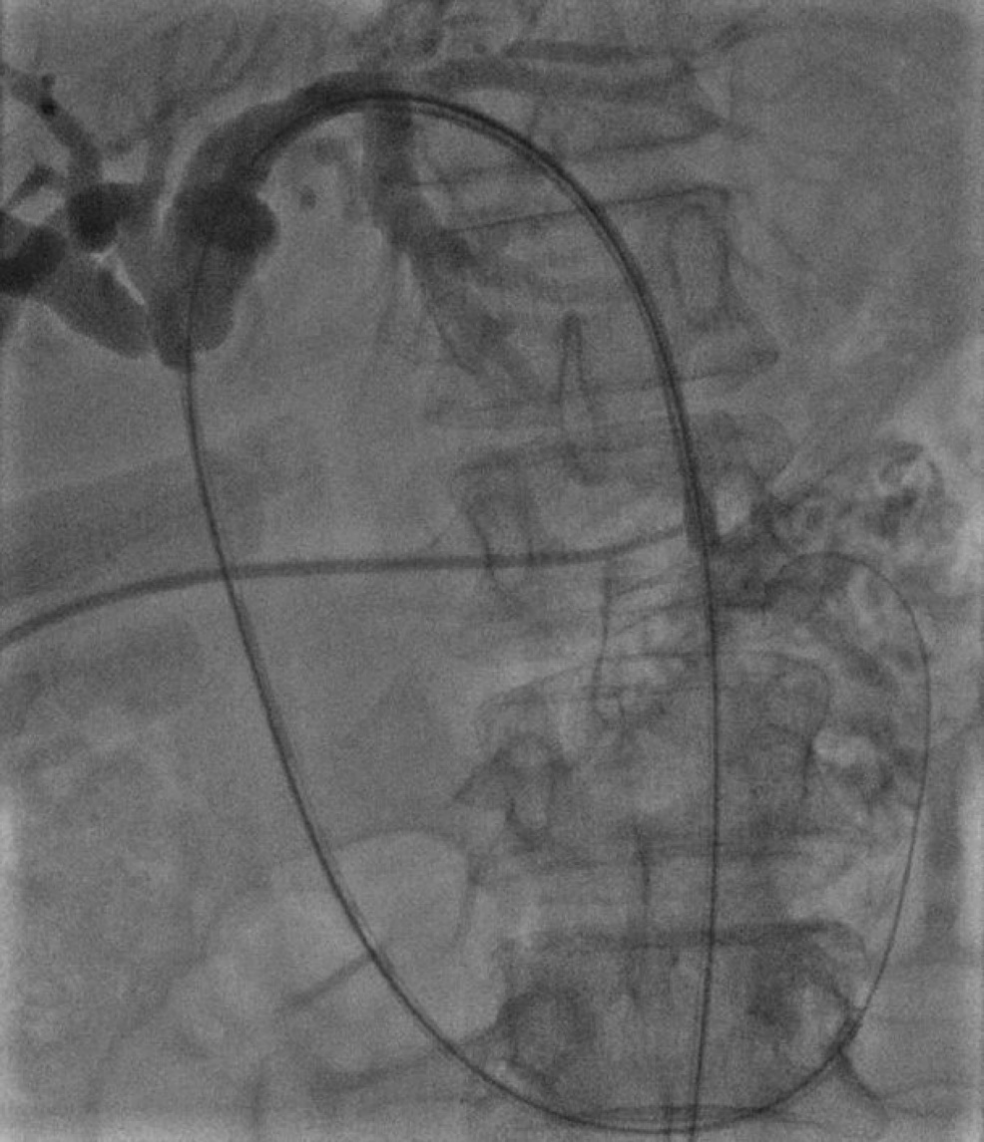
Percutaneous transhepatic cholangiogram and cannulating through the stricture into the common bile duct and duodenum.

**Figure 4:**
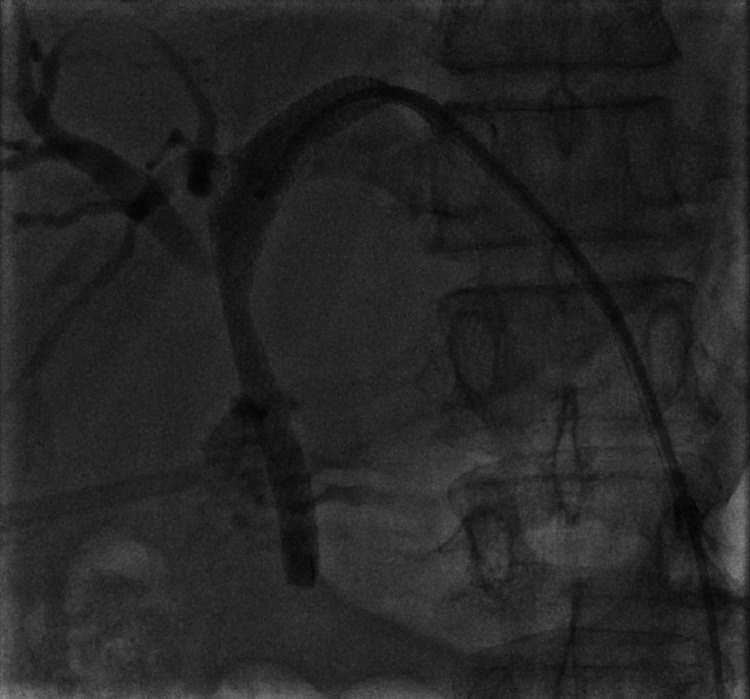
DSA image of placed self-expanding metallic stent (7FG × 10 cm) and contrast flow distal to the stricture. DSA: digital subtraction angiography

Percutaneous transhepatic biliary stenting was chosen as the next therapeutic option for the patient. The guidewire was negotiated through the previous EBD, bypassing the narrowed part of the hepatic duct under the guidance of digital subtraction angiography (Figure 3), and an uncovered self-expanding metallic stent (7FG × 10 cm) was placed (Figure 4).

Post-procedure and on the same day, the patient started to deteriorate. He developed abdominal pain, reduced urine output, and fever. He had tenderness and guarding of the abdomen, tachycardia, and his blood pressure dropped to 90/60 mmHg. IV meropenem and metronidazole were immediately initiated.

Urgent imaging studies (ultrasonography and non-contrast CT of the abdomen) revealed free fluid collection in the subdiaphragmatic and subhepatic spaces and in the pelvic cavity with a 1 cm part of the stent displaced outside the liver capsule. An ultrasound-guided pigtail drain was inserted into the subdiaphragmatic space where there was a potential bile leak. However, the pigtail drainage did not improve the patient’s clinical condition, and it was decided to perform a diagnostic laparoscopy and washout.

**Figure 5:**
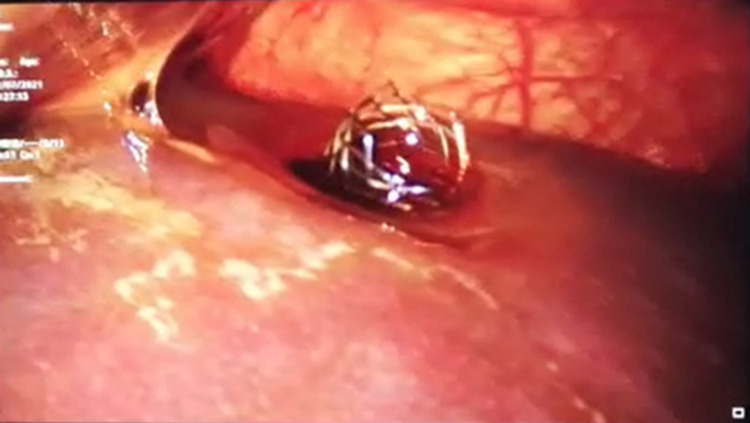
Laparoscopic image of the displaced stent on the liver surface.

On the same day at midnight, diagnostic laparoscopy was performed. Visual inspection revealed bile in the peritoneal cavity with multiple pockets of fluid in the subhepatic space, subdiaphragmatic space, and pelvic cavity. In addition, there was a 1 cm extracapsular displaced part of the stent with an active bile leak (Figure 5). We failed to push the stent inside the liver; hence, the extracapsular part of the stent was cut and removed by pulling the stent using a suture-cutting laparoscopic scissor. A 2 cm part of the stent was retrieved from the cut section, and the remaining stent was retracted into the liver parenchyma. Subsequently, the leak ceased. The pockets of fluid were drained, and the peritoneal cavity was thoroughly washed with normal saline. Four drains were inserted into the sites (subhepatic, subdiaphragmatic, and pelvic) where fluid was collected and the site of the bile leak.

Intraoperatively, the patient developed hypotension. He was started on noradrenaline infusion and transfused blood. Postoperative care via close observation was provided in the intensive care unit, the drains were separately monitored, and the patient was mobilized as quickly as possible. The drains were removed once there was minimal to no output. The subdiaphragmatic drain was removed first, followed by other drains; finally, the pelvic drain was removed on postoperative day five. Transabdominal ultrasonography confirmed no residual fluid. The bilirubin levels and inflammatory markers returned to normal. The patient clinically improved and was discharged on postoperative day eight. On follow-up, he was doing well and awaiting laparoscopic cholecystectomy.

## Discussion

Biliary tract injuries occur most often due to iatrogenic causes [3,5]. Bile leak with or without peritonitis is a potentially life-threatening event unless recognized and treated early [5]. Patients with bile leaks due to iatrogenic causes usually only have a small leak and develop symptoms insidiously [6], unlike our patient who developed a significant leak and deteriorated rapidly. High clinical suspicion, biochemical and hematological investigations, and imaging such as transabdominal ultrasonography and CT remain the mainstays of diagnostic aids in biliary leak [6]. Ultrasound-guided drain placement can be attempted in patients with minimal leak [3]. However, patients with a continuous leak similar to ours require leak control and peritoneal washout. In addition, early intervention reduces the risk of peritonitis. Displaced stents and parts outside the liver but within the peritoneal cavity can be cut and removed safely. To our knowledge, no cases such as ours have been reported in the literature. Therefore, the experiences we have reported in this paper can be used to guide clinicians in the treatment of similar cases.

## Conclusions

Active biliary leakage should be considered in rapidly deteriorating patients following any procedure in the biliary tract. Measures should be taken to control the leakage, the peritoneal cavity should be washed out, and drains should be placed in cases of significant biliary leaks. Compared with more invasive open surgeries, laparoscopy plays a crucial role in managing patients with fewer morbidities.

## References

[1] Dumonceau JM, Heresbach D, Devière J, Costamagna G, Beilenhoff U, Riphaus A (2011). Biliary stents: models and methods for endoscopic stenting. Endoscopy.

[2] Kaw M, Singh S, Gagneja H (2003). Clinical outcome of simultaneous self-expandable metal stents for palliation of malignant biliary and duodenal obstruction. Surg Endosc.

[3] Sharma M, Prabha V, Devaraju S (2020). Injury to biliary tract during percutaneous nephrolithotomy: minimally invasive management of a dreadful complication. J Endourol Case Rep.

[4] Castagnetti M, Houben C, Patel S (2006). Minimally invasive management of bile leaks after blunt liver trauma in children. J Pediatr Surg.

[5] Patel SR, Nakada SY (2010). Biliary peritonitis after percutaneous nephrolithotomy: case studies and management concerns. J Endourol.

[6] Massoumi H, Kiyici N, Hertan H (2007). Bile leak after laparoscopic cholecystectomy. J Clin Gastroenterol.

